# Supplementary material for: Preparation of Ce–Mn Composite Oxides with Enhanced Catalytic Activity for Removal of Benzene through Oxalate Method
Source: Nanomaterials (Basel). 2019 Feb 3;9(2):197. doi: 10.3390/nano9020197 (PMC6409587; doi:10.3390/nano9020197)
Supplement: Supplementary file 1 [file nanomaterials-09-00197-s001.pdf]

Preparation of Ce–Mn composite oxides with enhanced catalytic activity for removal of benzene through oxalate method

Min Yang[1,⊥], Genli Shen[2,⊥], Mi Liu[2], Yunfa Chen[3], Zhen Wang[2,*] and Qi Wang[2,*]

Supporting Information

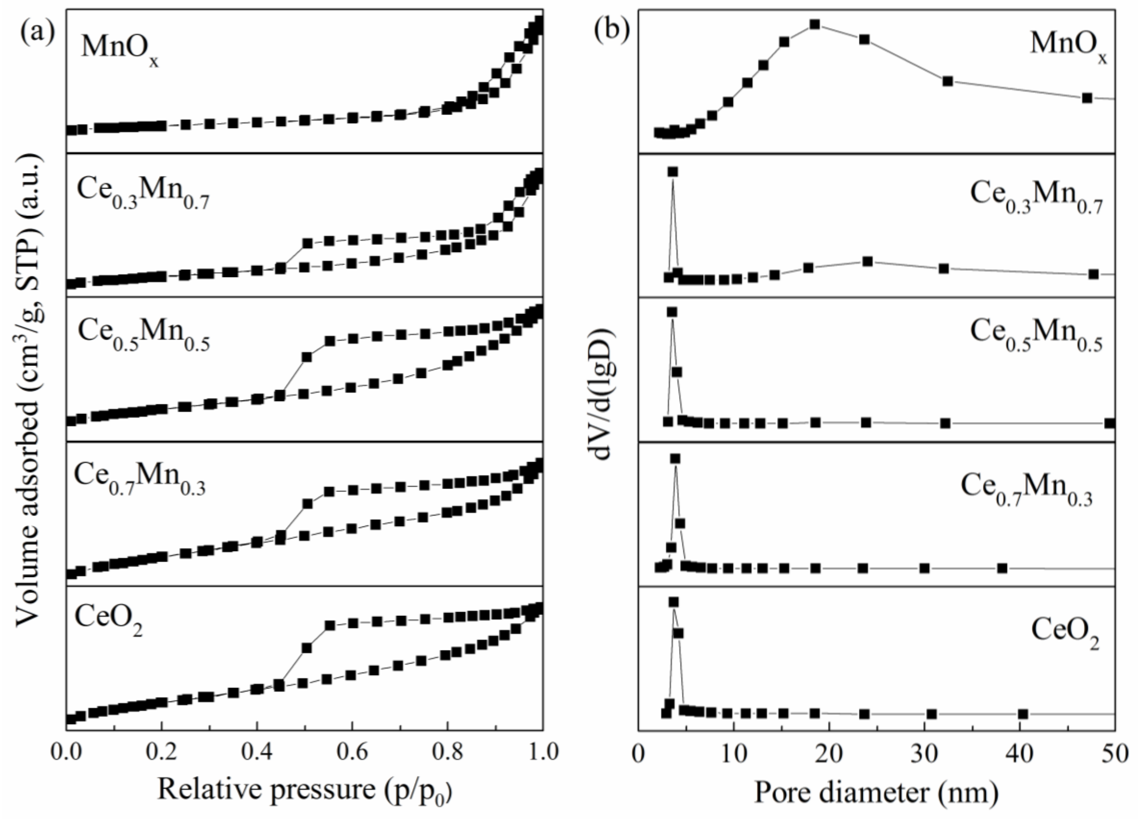

Figure 1. N_2 adsorption-desorption isotherms and pore-size distribution of CeO_2, MnO_x and Ce_xMn_{1-x} catalysts.

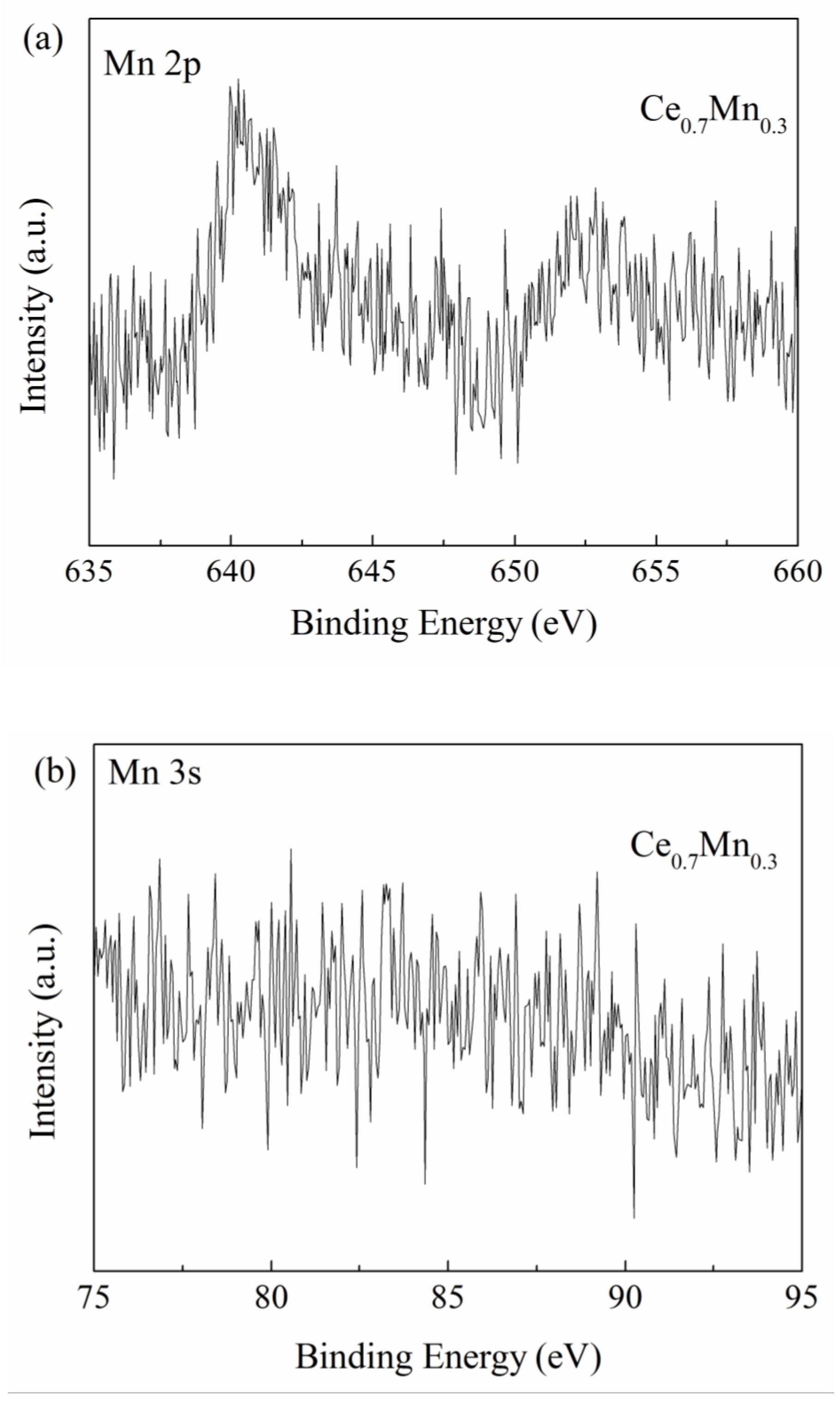

Figure S2. X-ray photoelectron spectra in the Mn 2p (a) and Mn 3s (b) regions for the $Ce_{0.7}Mn_{0.3}$catalysts.
